# Need-based resource allocation: different need indicators, different results?

**DOI:** 10.1186/1472-6963-9-122

**Published:** 2009-07-21

**Authors:** George Kephart, Yukiko Asada

**Affiliations:** 1 Department of Community Health and Epidemiology, Dalhousie University, Centre for Clinical Research, 5790 University Avenue, Halifax, Nova Scotia, B3H 1V7, Canada

## Abstract

**Background:**

A key policy objective in most publicly financed health care systems is to allocate resources according to need. Many jurisdictions implement this policy objective through need-based allocation models. To date, no gold standard exists for selecting need indicators. In the absence of a gold standard, sensitivity of the choice of need indicators is of concern. The primary objective of this study was to assess the consistency and plausibility of estimates of per capita relative need for health services across Canadian provinces based on different need indicators.

**Methods:**

Using the 2000/2001 Canadian Community Health Survey, we estimated relative per capita need for general practitioner, specialist, and hospital services by province using two approaches that incorporated a different set of need indicators: (1) demographics (age and sex), and (2) demographics, socioeconomic status, and health status. For both approaches, we first fitted regression models to estimate standard utilization of each of three types of health services by indicators of need. We defined the standard as average levels of utilization by needs indicators in the national sample. Subsequently, we estimated expected per capita utilization of each type of health services in each province. We compared these estimates of per capita relative need with premature mortality in each province to check their face validity.

**Results:**

Both approaches suggested that expected relative per capita need for three services vary across provinces. Different approaches, however, yielded different and inconsistent results. Moreover, provincial per capita relative need for the three health services did not always indicate the same direction of need suggested by premature mortality in each province. In particular, the two approaches suggested Newfoundland had less need than the Canadian average for all three services, but it had the highest premature mortality in Canada.

**Conclusion:**

Substantial differences in need for health care may exist across Canadian provinces, but the direction and magnitude of differences depend on the need indicators used. Allocations from models using survey data lacked face validity for some provinces. These results call for the need to better understand the biases that may result from the use of survey data for resource allocation.

## Background

A key policy objective in most publicly financed health care systems is to allocate resources according to need. Many jurisdictions implement this policy objective through need-based allocation models. A number of countries (e.g., Australia, Norway, the United Kingdom), for example, employ need-based models to allocate health care resources among regions[1]. Though less common, need-based models are also used in determining capitation payments to enrolment-based provider groups[2]. The methods used to develop need-based models have advanced considerably since the initial work of the United Kingdom Resource Allocation Working Party in the mid-1970s [3]. Considerable challenges remain, however, from data availability to modelling strategies [4-6].

Key questions that any developers of need-based resource allocation models must ask is which need indicators to use, and whether the choice of need indicators and modelling strategies will produce plausible results in different settings. To date, no gold standard exists for the choice of need indicators, and thus models and the indicators they use vary considerably[4]. Demographic factors (e.g., age and sex) are routinely used as need indicators. Most models also include additional need indicators such as measures of health status (e.g., self-reported health), and socioeconomic status (e.g., income and education). Allocation models can employ individual-level data on resource use and need indicators, but because of data limitations (especially the lack of survey data with adequate sample size by region), they are often based on need indicators and models for small areas (e.g., premature mortality rates or rates of morbidity)[5,7]. Survey data, however, are increasingly used as a key data source due to the range of variables available and flexibility in modelling[5,8,9].

In the absence of a gold standard, comparing and assessing the face validity of allocations from models using different need indicators is important. For example, do we obtain similar results when using different need indicators? Do the allocations estimated by different types of models make sense given what we know about differences in the health status and mortality patterns in regional populations? Despite the profound policy implication of this question, little direct comparison of allocations arising from models using different need indicators exists. The lack of data that easily allow such comparison is one reason for the paucity of such studies.

Canada offers an interesting setting and rich data sources to compare resource allocations from models based on different need indicators and to assess their face validity. Canadian provinces are geographically dispersed, display considerable variation in socioeconomic status and health, and thus can be expected to vary in the need for health services. Moreover, these differences may have important implications for how health care is financed and delivered. Health care in Canada is regulated nationally but is administered through thirteen different provincial and territorial health insurance plans and delivery systems. The Canada Health Act subjects these systems and plans to common principles and requirements with the goal of ensuring that all residents have reasonable access to needed health services without financial or other barriers. Provincial health systems are financed through a combination of provincial revenue and federal funding (provided to the provinces through health specific and general transfer payments). However, the fiscal capacity of provinces to deliver health care varies considerably as a result of differences in tax bases. However, need-based approaches are not used to allocate federal funding, and with the exception of Kephart et al [10], little previous work has examined relative need for health services between Canadian provinces. Kephart et al. demonstrated that differences in expenditure need may be important contributors to fiscal inequities among provinces. Therefore, consideration of differences in provincial health care need and incorporation of those differences into the allocation of transfer payments is needed.

Canada has rich sources of survey data that are well suited to estimating and comparing alternative need-based resource allocation models. Most notably, the Canadian Community Health Survey (CCHS) provides repeated cross-sections documenting the health and health care utilization of Canadians. This survey contains a large sample size designed to provide provincial and sub-provincial estimates of health status and health services use, allowing comparison of resource allocation models using different need indicators.

The objective of this study was to assess the consistency of estimates of per capita relative need for health services based on different need indicators across Canadian provinces and assess their face validity. While the primary contribution of this paper is methodological, our analysis will inform discussion on equitable allocation of Canadian federal transfer payments to provinces to support provincial health programs.

## Methods

### Overview

The standard procedure for need-based resource allocation, which we use for our analysis, proceeds in two stages[5,8,11,12]. The first modeling stage is to estimate "standard levels of resource use" by different levels of need. That is, it estimates the average relationship between need and resource use in the population. The premise of this standard is the principle of sharing available resources in a consistent way across the population given the distribution of need[12]. The second allocation stage applies this standard to the population characteristics of each region. Relative per capita resource allocations thus reflect differences in the number and distribution of need attributes, not regional differences in actual levels of utilization. Populations with a higher share of persons with greater need will be assigned a higher per capita allocation. Below we explain these two stages in detail.

#### Stage 1: Modeling utilization and predicted use based on need

The first stage employs models using observed utilization as the dependent variable and proceed in two steps. The first step, using individual-level data representative of the target population, estimates a model of the form:

(1)

where *y*_*i *_is the utilization for individual i, *A*_*ij *_is a vector of age-sex dummies, *X*_*ik *_is a vector of additional needs indicators, *Z*_*il *_is a vector of non-need determinants of utilization, and the *R*_*im *_are dummy variables for regions. The coefficient vectors *β*, *λ*, *γ*, *δ*, and *φ *estimated from this model describe the average utilization in the population by age-sex group, additional need indicators and non-need factors. The δ captures interactions between need indicators and age (other interactions are possible, but age interactions are the most plausible). The coefficients *φ *capture unmeasured need and non-need variables associated with the regions[5]. Ideally, the utilization model should include both need and non-need determinant of utilization in order to get unbiased estimates of the coefficients of the need variables [5,9,13].

The second step computes, for a sample representative of the population and its allocation regions, the standard utilization levels based on need indicators alone. This standard resource requirement based on need is calculated as predicted values from equation 1, holding values of *Z *and *R *constant (at their means) so that need factors alone influence the predictions[5,9]. That is, the effects of non-need factors are purged using an equation of the form:

(1a)

where the  is the expected resources required for sample member *i *when *Z *and *R *variables are held constant across all regions, and the , , ,  and  are from equation 1.

#### Stage 2: Allocating to regions with the need-based model

Using the standard levels of resource use based on need from equation 2, the second stage estimates per capita resource need for residents of each allocation region (*N*_*r*_) as follows:

(2)

where the *w *is the survey sample weight for each individual. Survey data used for this purpose should be appropriate in sample size and design for making regional estimates. Estimates of per capita need for each region can then be used, along with population counts, to calculate regional allocations. For a need indicator to have an impact on allocation using this approach, it has to be a significant predictor in equation 1 and be differentially distributed across regions.

### Approaches

Following the standard procedures for need-based resource allocation described above, we estimated relative per capita need for general practitioner, specialist, and hospital services by province using the following two approaches that incorporate a different set of need indicators:

• Approach 1 estimated relative per capita need for health services based on age, sex and age-sex interactions, with no adjustment for non-need factors. Approach 1 is considered a fundamental need adjustment and used widely[4].

• Approach 2 estimated relative per capita need for health services based on demographics, multiple indicators of need (e.g., measures of health status, *X*), and interactions between need variables and age (*A *and *X*), with adjustment for non-need factors (*Z*). Approach 2 is similar in many respects to methods currently used in the United Kingdom, and proposed in Ontario for estimating relative need for home care services[8,9,14].

### Data sources

Data on health care utilization, demographics, socioeconomic status, and health status came from the 2000/2001 Canadian Community Health Survey (CCHS)[15]. The CCHS is a cross-sectional survey that has been implemented every two years since 2000/2001. Although more recent years of data are available, the 2000/2001 CCHS offers the largest sample size for the greatest number of variables, which is critical for the complex modeling strategies we employed. The CCHS collects information on health determinants, health status, and health care utilization by personal or telephone interview. It uses a multi-stage stratified cluster sample design and collects information from one or two persons aged 12 years or older in each selected household in all provinces and territories. Less populous regions were oversampled to provide reliable provincial and sub-provincial estimates. Excluded from the sampling frame are people living on Indian Reserves and in institutions, Canadian Forces Bases, and some remote areas, who are estimated to account for 2% of the Canadian population aged 12 years or older. Thus, some groups that likely have high need for health services are omitted. The target total sample size for the 2000/01 CCHS was 133,300, and the response rate was 84.7%. We limited our analysis to adults (age 20 and over) who reside in the 10 provinces. We believe that modeling standard health care need for children and residents of the territories requires separate models due to their unique needs, and the in the case of the territories, organization of health care services. The CCHS does not have adequate data to support models for these populations due to exclusions and sample size. The sample size for all analyses presented in this paper was 111,249. Provincial sample sizes for our analysis ranged from a minimum of 3,264 in the least populous provinces to a maximum of 34,189 in Ontario.

### Variables

The dependent variables for the two approaches were based on self-reported numbers of general practitioner, specialist, and hospital use for the past 12 months. While research shows that data on self-reported health care use is subject to some bias, it has also been found to have a high level of agreement with clinical data on use[16]. The use of general practitioners was the number of visits to family doctor or general practitioners. The use of specialists was the number of visits to specialists such as surgeons, allergists, orthopedists, gynecologists, or psychiatrists. The use of hospital was the number of overnight stays as a patient in a hospital, nursing home, or convalescent home. For each type of utilization, we constructed two dependent variables. The first was a binary variable indicating use versus on-use, and the second was a variable indicating the number of visits or the number of overnight stays for those who had at least one contact.

For Approach 1, we only used age, sex and age-sex interactions as the independent variables. For Approach 2, we included multiple independent variables, in addition to age and sex, as indicators of need and non-need determinants of health care utilization [Additional file 1].

Indicators of need included age, sex, major health risk factors (e.g., smoking status) as well as a variety of self-reported measures of health status and chronic diseases. In addition, we included interactions between age and the specific chronic conditions listed in [Additional file 1] We included several types of non-need indicators of utilization. Measures of socioeconomic status (education, household income and homeownership) and minority status were included to adjust for differences in access to care. Estimated differences in health care utilization by socioeconomic status, after adjustment for direct measures of health status, have been widely used in the literature as a means of estimating inequities in access to health care services [17-20]. We also included several variables as non-need variables to adjust for use of services that could be substituted for physician services, such as use of alternative health care services, self-help group participation, and sense of belonging to the community. We did not have direct measures of supply, or measures of access to health services that we could include as non-need variables. At present, comparable measures of supply for small areas are not publicly available. We also included fixed effects (dummy variables) for health regions in the models. These were based on 105 CCHS defined health regions which approximate provincial planning districts. These dummies were included to capture unmeasured supply, access and need, and should help to reduce omitted variable and endogeneity bias in estimates of other effects in the models[5].

Most of the variables included missing values. We imputed missing values for the Health Utilities Index (about 1.4% of the sample) using regression imputation with variables whose spearman correlation coefficient with the Health Utilities Index was greater than 0.10 (age, overweight, activities of daily living, instrumental activities of daily living, education, use and non-use of general practitioners, number of visits to general practitioners, number of hospital stays, self-perceived health, number of chronic conditions, arthritis, high blood pressure, heart disease, and eye problem). When possible, we assigned missing values to logical existing categories. For example, missing values for self-reported specific chronic conditions were coded as not having the condition. For variables with multiple categories, we created an additional missing category. For example, we assigned those who did not have income information to a missing category (about 10% of the sample) and those who were currently in school and those who did not have education information in separate categories (about 1.48% and 1.09% of the sample). We also conducted sensitivity analyses with missing data excluded and found that this did not affect study conclusions.

### Analysis

For both approaches, we employed the standard two-stage procedure described above. We first estimated regression models, corresponding to equation 1, to estimate standardized utilization of each of three types of health services (general practitioner, specialist, and hospital services). As is now common practice, we used two-part models to estimate the utilization models [21-23]. Part 1 examines the dichotomous decision of use versus non-use of services, and we used logistic regression. Part 2 examines the amount of use conditional on being a user, and we used zero-truncated negative binomial regression[21]. We estimated regression coefficients using maximum likelihood estimation. Our models were unweighted so that the estimates of coefficients for need variables were not disproportionately determined by the most populous areas of the country. However, we compared our results with weighted models, and found that it did not impact the conclusions of our study.

We computed estimates of standard health services requirements based on need for each individual in the sample, the  in equation 2, as the product of the probability of use, derived from part 1 of the two part regression models, and the amount of use conditional on being a user, derived from part 2. Coefficients for non-need variables were fixed at their means. We used Stata version 10 for all stages of the analysis[24].

We computed per capita need for each type of health service (the *N*_*r *_in equation 3) as the sample weighted mean of expected level of health services for survey respondents in each province. We then expressed the estimated per capita need for health services in each province relative to the Canadian average, using:

(4)

Where *N*_*r *_is the estimated per capita utilization for province *r*, and *N*_*CA *_is the estimated per capita utilization for Canada. This facilitates interpretation by expressing provincial need as percent deviations from the Canadian average.

### Comparison with premature mortality

To check face validity of per capita relative need for the three health services estimated by Approaches 1 and 2, we compared the results with premature mortality across provinces. We defined premature mortality as the Standardized Mortality Ratio (SMR) for the population under age 75 and obtained data necessary to calculate it for each province in 2001 from Statistics Canada through E-STAT and CANSIM. To assist the comparison, we expressed premature mortality in each province as percent deviations from the Canadian standard. Although premature mortality in itself does not suggest need for specific health services, we expected that premature mortality and per capita relative need estimated by the two approaches would agree in direction.

## Results

### Expected relative per capita need for general practitioner services

**Figure 1:**
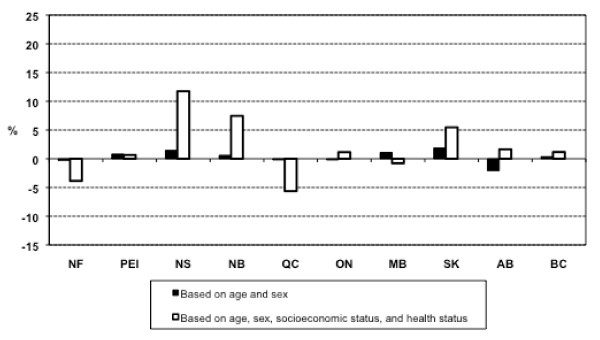
**Estimates of relative per capita need for general practitioner services**. NF: Newfoundland and Labrador; PEI: Prince Edward Island; NS: Nova Scotia; NB: New Brunswick; QC: Quebec; ON: Ontario; MB: Manitoba; SK: Saskatchewan; AB: Alberta; BC: British Columbia.

Additional file 2 and Additional file 3 show the results of regression models for general practitioner services corresponding to equation 1 and Approach 2. Figure 1 shows results of expected relative per capital need for general practitioner services estimated by the two approaches. Both approaches suggested that expected relative per capita need for general practitioner services was not uniform across provinces. The estimated range of unequal need was substantially smaller in Approach 1 (ranging from -2.07% in Alberta to 1.90% in Saskatchewan) than for Approach 2 (ranging from -5.63% in Quebec to 11.77% in Nova Scotia). Consideration of only age and sex as need indicators (Approach 1) resulted in modest inter-provincial differences in estimates of per capita need for general practitioner services. Consideration of health status indicators, in addition to age and sex, resulted in much greater inter-provincial differences in need.

### Expected relative per capita need for specialist services

**Figure 2:**
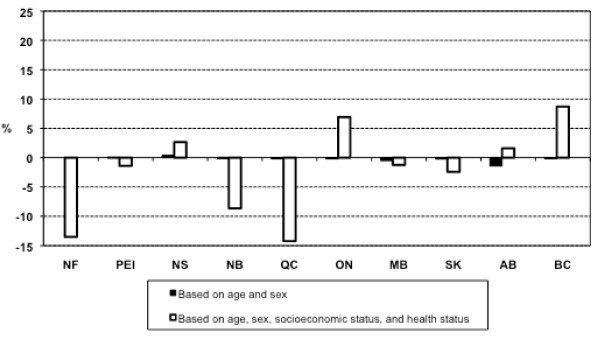
**Estimates of relative per capita need for specialist services**. NF: Newfoundland and Labrador; PEI: Prince Edward Island; NS: Nova Scotia; NB: New Brunswick; QC: Quebec; ON: Ontario; MB: Manitoba; SK: Saskatchewan; AB: Alberta; BC: British Columbia.

Additional file 4 and Additional file 5 show the results of regression models for specialist services corresponding to equation 1 and Approach 2. Figure 2 shows estimates of relative per capital need for specialist services based on the two approaches. Results for specialist services were similar to the results for general practitioners. Both approaches again suggested differential need for specialist services across provinces. The range of differences estimated by Approach 1 (ranging from -1.42% in Alberta to 0.40% in Nova Scotia) was even smaller for specialist than general practitioner services. Thus, consideration of age and sex alone as need indicators yielded only small differences in estimates of need. However, the addition of other indicators of need (Approach 2) resulted in large and substantial inter-provincial differences in estimates of need (ranging from -14.24% in Quebec to 8.73% in British Columbia).

### Expected relative per capita need for hospital services

**Figure 3:**
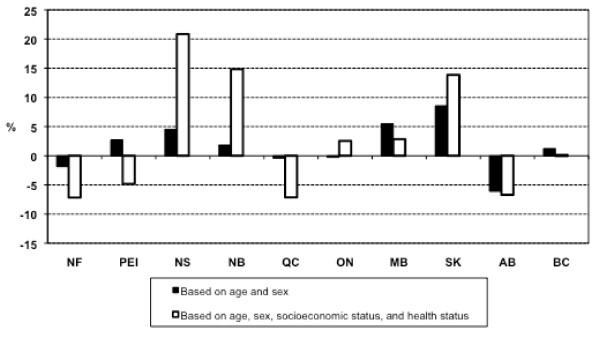
**Estimates of relative per capita need for hospital services**. NF: Newfoundland and Labrador; PEI: Prince Edward Island; NS: Nova Scotia; NB: New Brunswick; QC: Quebec; ON: Ontario; MB: Manitoba; SK: Saskatchewan; AB: Alberta; BC: British Columbia.

Estimated regression parameters for hospital services and Approach 2 are shown in Additional File 6 and Additional file 7. Figure 3 shows relative per capital need for hospital services estimated by the two approaches. Compared with the results for physician services, the estimated inter-provincial differences in per capita need for hospital services were greater. Consideration of age and sex alone as need indicators for the use of hospital services (Approach 1) resulted in large interprovincial differences in estimates of per capita relative need. This was in stark contrast to the results for general practitioner and specialist services. The magnitude of differences in need for hospital services for Approach 1 ranged from -6.10% for Alberta to 8.58% for Saskatchewan. As with the results for physician services, the inclusion of additional indicators of need in Approach 2 resulted in substantially larger inter-provincial differences in per capita need. The magnitude of differences in need for hospital services ranged from -7.16% (Newfoundland) to 20.86% (Nova Scotia) in Approach 2.

### Comparison with premature mortality

**Figure 4:**
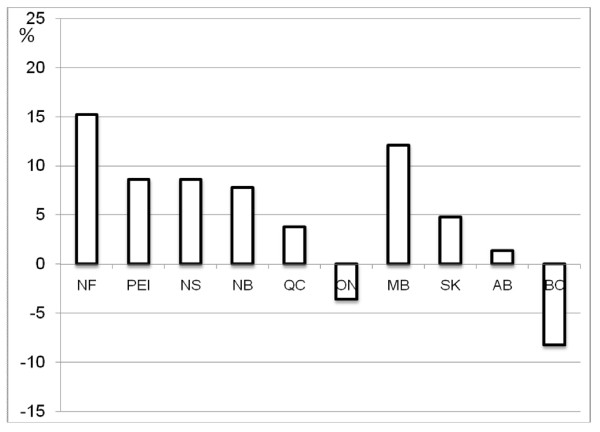
** Premature mortality**. NF: Newfoundland and Labrador; PEI: Prince Edward Island; NS: Nova Scotia; NB: New Brunswick; QC: Quebec; ON: Ontario; MB: Manitoba; SK: Saskatchewan; AB: Alberta; BC: British Columbia.

Provincial per capita relative need for the three health services did not always indicate the same direction of need suggested by premature mortality in each province (Figure 4). In particular, Approach 1 and 2 suggested Newfoundland had less per capita need than the Canadian average for all three services, but it had the highest premature mortality in Canada. Higher than average premature mortality for Quebec was also inconsistent with its lower estimates of need from both approaches. Among generally inconsistent need suggested by the two approaches and premature mortality, Nova Scotia was a clear exception; by any measures Nova Scotia was a province with greater need than the Canadian average.

## Discussion

To assess the consistency of estimates of per capita relative need for health services based on different need indicators, this study estimated differences in per capita need for health services between Canadian provinces using two approaches. These approaches, similar to allocation models used in the past, employed different sets of need indicators. Our primary conclusion is that different approaches yielded different and inconsistent results. For example, models only incorporating age and sex (Approach 1) estimated much smaller inter-provincial differences in per capita need than models that also employed measures of health status (Approach 2). These results are consistent with a study of capitation formulae for physician payments in Ontario, which also showed that adding health status, socioeconomic characteristics, and mortality to formulae that employ only age and sex modify allocations[2]. Also, the effect of age and sex on estimates of resource need varied by type of health care service. For general practitioner services, and especially specialist services, age and sex alone only resulted in small differences in relative per capita need. However, for hospital services, accounting for age and sex alone resulted in large inter-provincial variation in per capita need.

Comparison with the two approaches with premature mortality further complicated the picture. The direction of need suggested by the two approaches and premature mortality are often inconsistent. For example, indicators of health status from a survey, including measures of self-reported health status and self-reported chronic conditions, suggested Newfoundland and Quebec had lower per capita need than the Canadian average for all services, while premature mortality suggests they should have higher need. In addition to having the highest premature mortality rate, Newfoundland also has the lowest life expectancy among Canadian provinces. We explored using different mixes of need indicators in Approach 2 and found the counterintuitive results for Newfoundland from Approach 2 to be robust. No single need indicator accounts for the lower estimated need for Newfoundland. The low estimated need for Newfoundland results from higher than average health status, and lower rates of major chronic conditions than most other provinces. Ontario and British Columbia had a similar discrepancy, but smaller and in the opposite direction.

While few would advocate the use of premature mortality ratios alone as a need indicator for allocation, it is a meaningful measure of health based on reliable data and is based on fundamentally different types of data sources. It thus provides an interesting "reality" check on allocation models. Inconsistencies between need indicated by survey-based and mortality related indicators, at the least, call for further investigation of what exactly these indicators are measuring, and further efforts to validate the allocations resulting from complex resource allocation models.

A number of factors may affect the validity of allocation models similar to Approach 2. Self-report errors or sampling error may affect validity. However, we believe that a bigger validity threat is that the prevalence of chronic health conditions and health states may, in some populations, be a poor indicator of need. The prevalence of self-reported conditions in survey data depends on the incidence rate of persons developing conditions, the average waiting time to diagnosis, and the average duration of survival following diagnosis. For example, if the risk of developing lung cancer remained unchanged, medical advances increasing the survival (or leading to earlier detection) of patients with lung cancer would result in increased prevalence rates[25]. Approach 2 would thus estimate higher health care need as a result of such a change. It is possible, for example, that Newfoundland may have lower prevalence of chronic conditions and higher measures of health status in surveys as a result of later diagnosis of major chronic disease and shorter survival. This may be compounded by under-reporting of chronic conditions after diagnosis and more optimistic self-reports of health status. Interestingly, this issue would not affect morality rate or life-table derived measures of health status such as premature morality or life expectancy.

Clearly, the prevalence of chronic conditions is an important indicator of the demand for health services. Persons who drop dead from a heart attack without being diagnosed with heart disease don't show up as prevalent cases of heart disease in surveys. On the other hand, they place little if any associated demand on the health system. Conversely, persons who are diagnosed early and survive heart attacks are more likely to be prevalent cases of heart disease in surveys, and will likely use a high volume of services over many years. In this sense, prevalence of chronic conditions is a good indicator of need. Models based heavily on the prevalence of chronic conditions and health states allocate funds to treat current ill health, but neglect investments required to improve health through prevention, early diagnosis, and improved quality of health care[6]. Given that many allocation models currently in use employ the prevalence of health problems and health states as need indicators, further work to understand the contribution of delayed diagnosis and shorter survival to regional variations in prevalence is needed.

A second objective of this study was to inform debates around equitable allocation of federal transfer payments to provinces in Canada. The Canada Health Transfer is one of the largest federal transfer payment and provides a substantial component of the funding for provincial health care programs. The Canada Health Transfer is intended to support all physician and hospital services mandated under the Canada Health Act. The last federal budget calls for the Canada Health Transfer to be allocated to the provinces on an equal per capita basis (i.e., solely based only on population size). Our results suggest that provincial differences in per capita need vary, regardless of the approach used. We found that the largest estimated inter-provincial differences in need were for hospital services, which are the most expensive component of publicly financed services in Canada. These results confirm our earlier work [10]. This suggests that there may be substantial mismatch between the shares of federal funding provinces receive and their relative need for health services. This may contribute to inequalities in the use of health services between provinces. In fact, a recent study found that income-related inequality in the use of health services in Canada is due more to inequality between rich and poor provinces than to income equality within provinces[26].

However, the inconsistent results between the different approaches present major challenges to policy makers who may wish to consider need in allocating health care resources. Further investigation is clearly necessary regarding reasons for the inconsistency. Policy implementation of need-based funding between provinces will require plausible and acceptable models. The inconsistent results also present a typical dilemma in health services research and policy: how much evidence is enough to implement or change policy? One might consider that the proposed equal per capita approach to allocating federal health transfers in Canada is an extreme answer.

This study has important limitations. First, our ability to adjust for important non-need determinants of utilization was limited. Purging the effects of need factors on utilization of bias resulting from non-need determinants is critical to estimating need from utilization data[5,18]. We lacked measures of supply of health services, as well as direct measures of access to care, and thus bias in the effects of need indicators are possible. Second, the effects of need indicators may also be biased by "endogeneity" as a result of the effects of health care utilization on need indicators [6,10]. However, our inclusion of fixed effects for health regions should have reduced both types of bias to the degree that supply and access effects are clustered within districts. Third, health care utilization was measured by self-report, which is subject to recall bias[16,27,28]. Ideally, self-reported health care utilization should be replaced by administrative data, but this is not currently possible using Canadian data. Finally, the standard errors in our analysis did not fully account for the complex sample design of the CCHS. Replication methods such as the bootstrap or the jackknife would be preferred[29]. The public use version of the CCHS used for this study did not contain the necessary information to permit such procedures. However, it is unlikely that bootstrapped standard errors would alter our results. The study employed a large sample size, so effects are estimated with high precision.

## Conclusion

Substantial differences in need for health care may exist across Canadian provinces, but the direction and magnitude of differences depend on the need indicators used. Allocations from models based on demographics, health status, and other need indicators obtained from survey data lacked face validity for some provinces. Given the increasing use of survey data for resource allocation, these results call for the need to better understand the biases that may result from the use of survey data for resource allocation.

## Abbreviations

CCHS: Canadian Community Health Survey; AB: Alberta; BC: British Columbia; MB: Manitoba; NB: New Brunswick; NF: Newfoundland; NS: Nova Scotia; ON: Ontario; PEI: Prince Edward Island; QC: Quebec; SK: Saskatchewan; SMR: Standardized Mortality Ratio.

## Competing interests

The authors declare that they have no competing interests.

## Authors' contributions

GK and YA were both equally involved in designing the study, conducting the analysis, interpreting the results, and drafting the manuscript. Both authors read and approved the final manuscript.

## Production note

This article was amended post-publication. Figures 1-3 that were available between 18 August and 16 September 2009 were included in error. The correct versions of these figures are now present in the manuscript.

## Pre-publication history

The pre-publication history for this paper can be accessed here:



## Supplementary Material

Additional file 1 Table 1. Variables of Interest Click here for file

Additional file 2 **Full logistic regression model for use of general practitioner services (Approach 2)**. The data provided represent the statistical analysis of a wide-range of predictive factors on probability of use vs. non-use of general practitioner services Click here for file

Additional file 3 **Full zero-truncated negative binomial regression model for the quantity of general practitioner services used by subjects with at least one visit (Approach 2)**. The data provided represent the statistical analysis of a wide-range of predictive factors on intensity of use of general practitioner services Click here for file

Additional file 4 **Full logistic regression model for use of specialist services (Approach 2)**. The data provided represent the statistical analysis of a wide-range of predictive factors on probability of use vs. non-use of specialist services Click here for file

Additional file 5 **Full zero-truncated negative binomial regression model for the quantity of specialist services used by subjects with at least one visit (Approach 2)**. The data provided represent the statistical analysis of a wide-range of predictive factors on intensity of use of specialist services Click here for file

Additional file 6 **Full logistic regression model for use of hospital services (Approach 2)**. The data provided represent the statistical analysis of a wide-range of predictive factors on probability of use vs. non-use of hospital services Click here for file

Additional file 7 **Full zero-truncated negative binomial regression model for the quantity of hospital services used by subjects with at least one visit (Approach 2)**. The data provided represent the statistical analysis of a wide-range of predictive factors on intensity of use of hospital services Click here for file

## References

[B1] Rice N, Smith P (2001). Capitation and risk adjustment in health care financing: An international progress report. Milbank Quarterly.

[B2] Hutchison B, Hurley J, Birch S, Lomas J, Walter SD, Eyles J, Stratford-Devai F (2000). Needs-based primary medical care capitation: development and evaluation of alternative approaches. Health Care Management Science.

[B3] Advisory Committee on Resource Allocation (1998). A brief historyof resource allocation in the NHS 1948–98. Resource Allocation Working Paper.

[B4] Hurley J, Hutchison B, Giacomini M, Birch S, Dorland J, Reid R, Pizzoferrato G (1999). Capitation formulae for integrated health systems: A policy synthesis. McMaster University Centre for Health Economics and Policy Analysis Policy Commentary.

[B5] Gravelle H, Sutton M, Morris S, Windmeijer F, Leyland A, Dibben C, Muirhead M (2003). Modelling supply and demand influences on the use of health care: Implications for deriving a needs-based capitation formula. Health economics.

[B6] Asthana S, Gibson A, Moon G, Dicker J, Brigham P (2004). The pursuit of equity in NHS resource allocation: should morbidity replace utilisation as the basis for setting health care capitations?. Social science & medicine (1982).

[B7] Smith P, Sheldon TA, Carr-Hill RA, Martin S, Peacock S, Hardman G (1994). Allocating resources to health authorities: results and policy implications of small area analysis of use of inpatient services. BMJ (Clinical research ed).

[B8] Sutton M, Gravelle H, Morris S, Leyland A, Windmeijer F, Dibben C, Muirhead M (2002). Allocation of resources to English areas: Individual and small area determinants of morbidity and use of healthcare resources. Report to the Department of Health.

[B9] Hurley JHB, Buckely G, Woodward C (2004). Developing need-based funding formulae using individual-level linked survey and utilization data: an application to home care services in Ontario. Centre for Health Economics and Policy Analysis Working Paper.

[B10] Kephart G, Pennock M, Skedgel C, James A, Nicol K, Ross J, Rossler N (2000). Federal funding for health care: Are provinces getting their fair share?.

[B11] Birch S, Kephart G, Tomblin-Murphy G, O'Brien-Pallas L, Alder R, MacKenzie A (2007). Human resources planning and the production of health: A needs-based analytical framework. Canadian Public Policy.

[B12] Rice N, Smith PC (2001). Ethics and geographical equity in health care. Journal of Medical Ethics.

[B13] Sutton M, Carr-Hill R, Gravelle H, Rice N (1999). Do measures ofself-reported morbidity bias the estimation of the determinants of health care utilisation?. Social Science and Medicine.

[B14] Rice N, Smith PC (1999). Approaches to capitation and risk adjustment in health care: An international survey.

[B15] Statistics Canada (2003). Canadian Community Health Survey, public use microdata file.

[B16] Bhandari A, Wagner T (2006). Self-reported utilization of healthcare services: improving measurement and accuracy. Med Care Res Rev.

[B17] Asada Y, Kephart G (2007). Equity in health services use and intensity of use in Canada. BMC Health Services Research.

[B18] Birch S, Eyles J, Hurley J, Hutchison B, Chambers S (1993). Aneeds-based approach to resource allocation in health care. Canadian Public Policy.

[B19] Dunlop S, Coyte PC, McIsaac W (2000). Socio-economic status and the utilisation of physicians' services: Results from the Canadian National Population Health Survey. Social Science and Medicine.

[B20] Katz SJ, Hofer TP, Manning WG (1996). Physician use in Ontario and the United States: The impact of socioeconomic status and health status. American Journal of Public Health.

[B21] Cameron AC, Trivedi PK (1998). Regression analysis of count data.

[B22] Gerdtham UG (1997). Equity in health care utilization: Furthertests based on hurdle models and Swedish micro data. Health economics.

[B23] Pohlmeier W, Ulrich V (1995). An econometric model of the two-part decisionmaking process in the demand for health care. Journal of Human Resources.

[B24] StataCorp (2005). Stata statistical software: Release 9.0.

[B25] Bonneux L, Barendregt JJ, Nusselder WJ, der Maas PJ (1998). Preventing fatal diseases increases healthcare costs: cause elimination life table approach. BMJ (Clinical research ed).

[B26] Jimenez-Rubio D, Smith PC, Van Doorslaer E (2008). Equity in health and health care in a decentralised context: evidence from Canada. Health economics.

[B27] Bellon JA, Lardelli P, Luna JD, Delgado A (2000). Validity of self reported utilisation of primary health care services in an urban population in Spain. Journal of epidemiology and community health.

[B28] Reijneveld SA, Stronks K (2001). The validity of self-reported use of health care across socioeconomic strata: a comparison of survey and registration data. International journal of epidemiology.

[B29] Lohr SL (1999). Sampling: Design and analysis.

