# Supplementary material for: Need-based resource allocation: different need indicators, different results?
Source: BMC Health Serv Res. 2009 Jul 21;9:122. doi: 10.1186/1472-6963-9-122 (PMC2728712; doi:10.1186/1472-6963-9-122)
Supplement: Additional file 1 — Table 1. Variables of Interest [file 1472-6963-9-122-S1.doc]

Table 1. Description of independent variables
