# Supplementary material for: Need-based resource allocation: different need indicators, different results?
Source: BMC Health Serv Res. 2009 Jul 21;9:122. doi: 10.1186/1472-6963-9-122 (PMC2728712; doi:10.1186/1472-6963-9-122)
Supplement: Additional file 2 — Full logistic regression model for use of general practitioner services (Approach 2). The data provided represent the statistical analysis of a wide-range of predictive factors on probability of use vs. non-use of general practitioner services [file 1472-6963-9-122-S2.doc]

Table 2. Full logistic regression model for use of general practitioner services (Approach 2)
