# Supplementary material for: Need-based resource allocation: different need indicators, different results?
Source: BMC Health Serv Res. 2009 Jul 21;9:122. doi: 10.1186/1472-6963-9-122 (PMC2728712; doi:10.1186/1472-6963-9-122)
Supplement: Additional file 3 — Full zero-truncated negative binomial regression model for the quantity of general practitioner services used by subjects with at least one visit (Approach 2). The data provided represent the statistical analysis of a wide-range of predictive factors on intensity of use of general practitioner services [file 1472-6963-9-122-S3.doc]

Table 3. Full zero-truncated negative binomial regression model for the quantity of general practitioner services used by subjects with at least on visit (Approach 2)
